# Ecological Correlates of Differences in Mean Age at Death Across Nearly Extinct Cohorts: The Role of Dietary Habits

**DOI:** 10.3390/nu18071021

**Published:** 2026-03-24

**Authors:** Alessandro Menotti, Paolo Emilio Puddu, David R. Jacobs, Anthony Kafatos, Miodrag Ostojic, Hanna Tolonen

**Affiliations:** 1 Association for Cardiac Research, Via Voghera, 31, 00182 Rome, Italy; amenotti2@gmail.com; 2 EA 4650, Signalisation, Électrophysiologie et Imagerie des Lésions d’Ischémie Reperfusion Myocardique, Université de Normandie, 14000 Caen, France; 3 Division of Epidemiology and Community Health, School of Public Health, University of Minnesota, Minneapolis, MN 55455, USA; jacob004@umn.edu; 4 Department of Social Medicine, Preventive Medicine and Nutrition Clinic, University of Crete, 70013 Heraklion, Greece; kafatos@med.uoc.gr; 5 Institute for Cardiovascular Diseases “Dedinje”, 11040 Belgrade, Serbia; mostojic2011@gmail.com; 6 Department of Public Health and Welfare, Finnish Institute for Health and Welfare, 00300 Helsinki, Finland; hanna.tolonen@thl.fi

**Keywords:** age at death, predictors, ecological investigations, residential cohorts, traditional risk factors, diet, nutrition

## Abstract

**Objectives.** Our objective was to study the ecological relationship of many risk factors and personal characteristics with mean age at death (AD) after a 50-year follow-up of nearly extinct cohorts. **Material and Methods.** There were 16 cohorts totaling 12,763 middle-aged men enrolled in the Seven Countries Study (SCS), and 58 variables were measured, including traditional risk factors, dietary nutrition and anthropometric variables. A follow-up of 50 years allowed the use of AD as the end-point. Analysis included simple linear regression correlation and multivariate modelling using Principal Component Analysis and regression and Ridge regression. **Results.** Out of 58 variables, only 11 (10 nutrition-dietary items plus age) showed a significant linear correlation coefficient (R) ≥ 0.50 and a *p* value ≤ 0.05. Linear regression was computed by using as a predictor the dietary factor score derived from a Principal Component Analysis of the 11 significant variables, which were used as independent variables, whose coefficients were significantly related with AD, and the final R^2^ was 0.52. The Principal Component regression and Ridge regression documented the direct relationship of food groups of vegetable origin (including olive oil) with the AD and the inverse relationship for food groups of animal origin. **Conclusions.** A few variables, all related to diet and nutrition, were able to statistically explain about 50% of the different AD in 16 cohorts of men followed up with nearly until death. Other variables, including traditional cardiovascular disease risk factors, did not contribute in a significant way for this purpose.

## 1. Introduction

Age at death (AD) is a simple and old demographic metric that has recently been re-evaluated [1,2,3,4,5]. Traditionally, it is used in demography, exploiting national official mortality data to study increasing longevity, to estimate the modelling of age and its distribution in pediatric and sport medicine in relation with the occurrence of sudden and unexpected death in infants and athletes, and in forensic medicine with the purpose of estimating age at death using complex autopsy procedures.

There is a great interest in longevity that has been increasing worldwide in recent decades. Among many existing studies, some are devoted to modelling the population structure [6], others are interested in the changing social and health problems among the new elderly population [7], and others try to identify the possible origins of increasing longevity in dietary habits [8].

In this study, our interest instead deals with the attempt to explain differences in longevity, expressed by AD across cohorts of people of the same sex and age range and living in the same historical period, examined directly through multiple standardized procedures and criteria and followed up with until near death, with the purpose of identifying which entry characteristics explain differences in AD.

In field epidemiology, AD can be used only if the study population cohorts have reached death or nearly death (usually with less than 10% of survivors) since otherwise, its meaning is deformed, not interpretable and difficult to compare across cohorts. On the other hand, there are a few investigations in which participants were followed up with until the cohort’s death, like in a Japanese study, where autopsy was performed in almost all participants [9], but this was a clinical exercise of about 800 consecutive patients enrolled at hospital admission and not a population study.

The Seven Countries Study of Cardiovascular Diseases (SCS) started in 1958, enrolling 16 cohorts of middle-aged men, and has reached exceptionally long follow-up for mortality, approaching extinction, which has allowed for the study of AD from different points of view. So far, little effort has been made to explain the different levels of AD across the enrolled cohorts based on an ecological approach, and only one dedicated paper was published in this area [10]. In the present investigation, we used the largest number of possible determinants of different conceptual features to explain the relatively large difference in AD across the 16 cohorts.

## 2. Material and Methods

**Populations and Measurements.** The 16 cohorts enrolled and first examined between 1958 and 1964 were located in 7 countries: USA, Finland, the Netherlands, Italy, former Yugoslavia (separately for the federate nations of Croatia and Serbia), Greece and Japan, for a total of 12,763 men aged 40–59 years. Ten cohorts were located in rural communities, 1 in a fishing community, and 4 in defined occupational groups, and 1 was a random sample of a small town. Details can be found elsewhere [11,12]. At the baseline examination, a large number of risk factors and personal characteristics were measured in all men [11,12]. In the early phases of the study, a complex dietary survey was run in subsamples of each cohort, allowing for the recording of many food groups and the chemical measurement of some basic nutrients in portions of food taken in the homes of the participants [10]. More details and related analyses have been reported elsewhere [13,14,15].

For the purpose of this analysis, 58 variables were identified as possible determinants of AD differences: 13 traditional cardiovascular risk factors, all expressed as the average from all men measured in the entry examination of the study [11]; 20 simple food groups; 4 combined food groups; 13 nutrients; and 8 ratios of food groups or nutrients and Dietary Scores, all measured in the dietary study mentioned above (expressed as their average to represent each cohort) [10,11,12,15]. The full list of all 58 variables is reported in Table A1 of Appendix A. The list of the major variables used in this analysis, together with their units of measurement and their abbreviations mainly adopted in further tables, is reported in Table 1.

For the measurement procedures of the possible determinants, details can be found in the quoted references, but for the 11 determinants individually associated with AD, some details are reported below:

(a) Age (in years), measured in all men, approximated to the nearest birthday and expressed in years (average for each cohort);
(b) Energy intake, derived from the dietary study on subsamples of each cohort, using the list of food groups and chemical measurements of food samples, expressed in calories per day (average for each cohort);
(c) Intake of liquid fat (all kind of oils), fruit, olive oil, hard fats and the sum of all vegetable food groups consumed, derived from the dietary study on subsamples of each cohort, from the list of food groups, expressed in grams per day and adjusted for 1000 calories (average for each cohort);
(d) M/S ratio (monounsaturated/saturated fat ratio), derived from the dietary study on subsamples of each cohort and from the list of nutrients and chemical measurements (average for each cohort);
(e) MP/ST ratio (monounsaturated + polyunsaturated fat/saturated fat + trans-fat), derived from the dietary study on subsamples of each cohort and from the list of food groups and chemical measurements (average for each cohort);
(f) Dietary thrombogenicity index (THI) following the rules from Ulbricht and Southgate [16] involving 10 types of fatty acids, expressed in arbitrary units (average for each cohort);
(g) Dietary inflammation index (INF), derived from the dietary study using energy, 24 nutrients and 26 simple or combined food groups as described in the pertinent reference, expressed in arbitrary units [10] (average for each cohort).

Mortality data were systematically collected and coded. For the purpose of this analysis the time horizon for mortality and consequently AD was fixed after the first 50 years of follow-up. At that time, mortality data and AD were practically complete for 11 cohorts, while they were available for shorter follow-up periods for the other 5 cohorts. Therefore, we adopted the procedure already successfully tested in other analyses [17,18], whereby incomplete 50-year mortality rates and AD for cohorts with shorter follow-up periods were estimated using regression equations derived from the 11 cohorts with complete mortality data for 50 years. The regression included as dependent variables the rates and AD of cohorts with a 50-year follow-up and as independent variables the 25- or 45-year follow-up data of the same cohorts. The 50-year estimates for the 5 cohorts with shorter follow-up periods were obtained by incorporating the 25- or 45- year data of these cohorts into the equation.

Death rates were expressed as per 1000 person/year in 50 years, and AD was expressed in years and computed only for cases of death. In this way, all-cause mortality covered about 97% of the denominator.

**Statistical analysis.** A descriptive analysis was run and tabulated, presenting cohort death rates and AD. A univariate analysis was computed, producing simple linear correlation coefficients (R) of all possible correlates (n = 58) versus AD. A correlation matrix was calculated across the 11 variables that were significantly associated with AD in the univariate analysis.

Multivariate analysis was performed using various types of models and using AD as the end-point (dependent variable) and adopting the following steps:

1. We computed all possible Multiple Linear Regressions (MLRs) using a maximum of 5 variables for a total of 131 MLRs.
2. The majority of the above models presented multicollinearity problems; the models were discarded, and as a consequence, we adopted two different steps.
3. Principal Component Analysis (PCA) was conducted involving the 11 significant variables in the univariate analysis. Starting from the correlation matrix of the 11 variables, this procedure produced a factor score (called Dietary Score) that was used to predict AD in a subsequent linear regression (with a single independent variable).
4. Then, we used the Principal Component Regression and the Ridge regression, which smooth the collinearity problems. A limited number of variables were used in the models, based on arbitrary *a priori* choice and/or convenience.

## 3. Results

Table 2 reports the mean levels of all-cause mortality and AD for each of the 16 cohorts where a relatively larger variation was found for cohort death rate than for AD. The highest levels of AD were found in Crete (Greece), Belgrade (Serbia, former Yugoslavia) and Rome Railroad cohorts (Italy); the lowest levels were found in Slavonia (Croatia, former Yugoslavia) and in East Finland, with a difference of over 10 years between the extremes. Death rates were inversely correlated with AD, with an R value of −0.96 (*p* < 0.0001), suggesting that higher death rates led to lower AD.

The first screening, consisting of a univariate analysis with the computation of simple linear regression coefficients (R) of each possible determinant versus AD, showed that out of 58 variables, only 11 had R ≥ 0.50 (*p* ≤ 0.05) (Table 3). Those 11 variables were age at baseline, LIF, FRU, OLI, VEG, ENE, HAF, M/S ratio, MP/ST ratio, THI and INF. Further analysis was concentrated on these variables. Among the dietary nutrition factors, the estimate of partial correlation was limited to each of the first five variables with the highest Rs, while the other four played the role of confounders. With this approach, all variables lost their significance except LIF, suggesting the existence of strong correlations across the five variables. The correlation matrix of the 11 variables (Table 4) indicates the existence of high R values, mainly those involving some variables like VEG, INF, M/S and MP/ST.

The detailed levels of the 11 predictive variables (Table 5) in each of the 16 cohorts is not easy to read and interpret. However, important information is provided by the coefficient of variation (standard deviation expressed as % of average) that is larger than the average (more than 100%) for LIF, OLI, FRU and INF, indicating substantial differences across cohorts in these variables and being a prerequisite of the possibility that these variables might play valuable roles in the association with the end-point.

**Figure 1:**
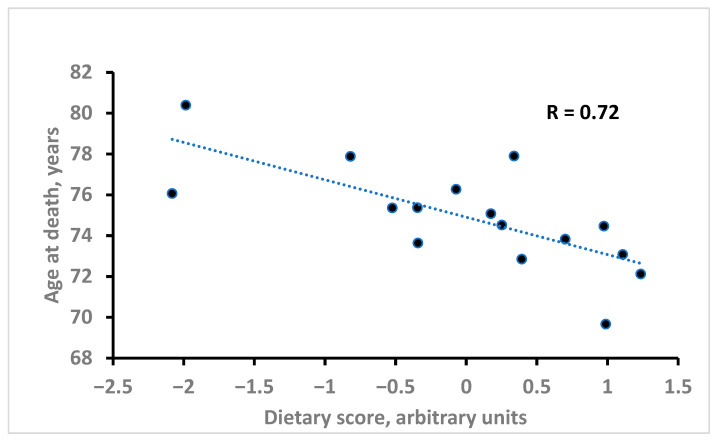
Relationship of Dietary Score with AD derived from univariate linear regression.

To overcome the difficulties in exploiting all the significant variables in the multiple regression models, we computed a model with AD as dependent variable and as predictive variables the dietary factor scores of each cohort derived from a Principal Component Analysis (PCA) of the 11 individually significant variables. The PCA factor scores (called Dietary Score) are reported in the right-end column of Table 3. The univariate regression model predicting AD showed that lower levels of score coefficients were associated with higher AD and vice versa. The original equation was AD = 74.91 − 1.83 Dietary Score, with R = 0.72 and R^2^ = 0.52. Figure 1 reports the correlation between Dietary Score and AD. In Table 3, there are two groups of Rs with the opposite algebraic sign, i.e., positive and negative, and the same two groups can be found in the columns of factor scores of the same table but characterized by opposite algebraic signs in a coherent way.

**Figure 2:**
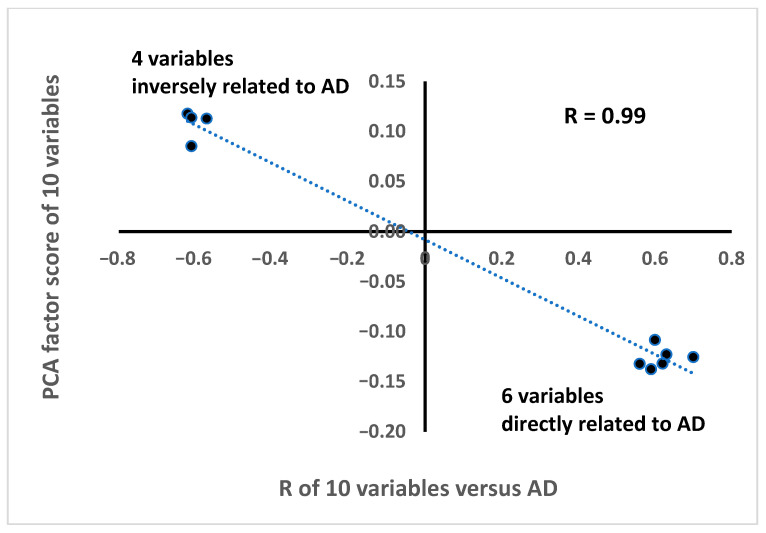
Correlation of the R value of the age at death of 10 variables (except AGE) versus the Dietary Score of the same variables. See Table 3 for the list of these variables (column R).

The singular fact is that the two groups were identified by the Rs that had an end-point as the dependent variable, while in the PCA, the same two groups (carrying opposite algebraic signs) were created only on the basis of their inter-correlation, without reference to an end-point. The R between univariate Rs of 10 variables and the correspondent Dietary Score was −0.99. This relationship is reported in Figure 2, where two small very compacted clouds of four and six points are located far away from each other, representing variables inversely and directly related to AD, respectively.

The Principal Component regression and the Ridge regression were run in parallel after an arbitrary choice of 6 among the 11 significant variables with significant linear correlation, i.e., 3 variables directly related and, separately, 3 variables inversely related to AD. Moreover, we considered models with two variables having opposite relationships with AD. In a preliminary check of the corresponding correlation matrix, the Variance Inflation Factor was less than 10, and no collinearity problems were detected in these groups. The Principal Component regression produced reasonable findings only when one component was omitted, and the findings are reported in the upper section of Table 6.

As a consequence of this constraint, and following a systematic search, the Ridge regression findings reported in the lower section of Table 6 are those most similar to the ones of the Principal Component regression, corresponding to the choice of k = 0.00001.

**Figure 3:**
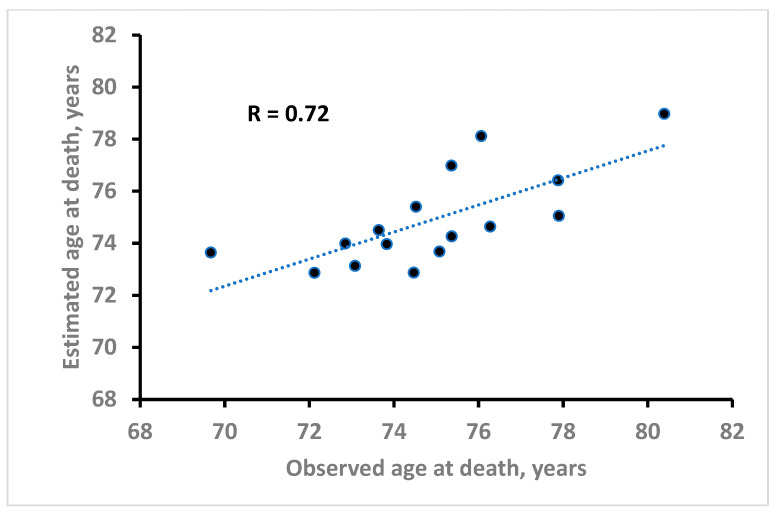
Relationship of observed versus estimated AD derived from a Ridge regression model based on 2 variables associated with AD in opposite ways.

The regression coefficients in all models’ approaches maintained the expected algebraic sign (positive for the variables directly related with AD and negative for the variables inversely related with AD), and this was true also in the models with two variables of opposite type. The *p* values, derived from the analysis of variance, were highly significant for the group comprising LIF, FRU and VEG, while the p values were only close to significant for the group comprising HAF, THI and INF. Larger *p* values were seen in the models with two variables of opposite type. As an example, the relationship of observed and estimated AD for the Ridge model dealing with LIF and HAF is reported in Figure 3 (R = 0.72).

## 4. Discussion

This analysis was based on a unique approach to the problem of longevity and its determinants that encompasses overall mortality, its components and its time of appearance using the metrics of AD. As a consequence, it cannot be applied to experimental studies. On the other hand, it is not guaranteed that the possible improvements in health care during the long follow-up period were similar across the various cohorts. However, the renowned high standard of the Finnish health care system did not prevent, mainly in the cohort of East Finland, the occurrence of low levels of AD.

Despite the exploration of many correlates of AD possibly capable of explaining cohorts’ differences in AD, the number of those producing significant correlations in the univariate analysis is rather few. Moreover, besides baseline age, 10 of 11 consisted of dietary nutrition items. The coefficient of age suggested that younger cohorts tended to reach higher AD, but this might be largely due to the relatively low entry age of some cohorts (like Belgrade and Rome Railroad) that also had very high or relatively high AD. The Belgrade cohort (university professors) was an outlier as it combined the lowest entry age with the second highest AD. In any case, in this ecological analysis, age at entry played an opposite role compared with that observed in analyses based on single individuals, where higher baseline age was associated with higher probabilities to reach high AD [19,20]. Moreover, the role of entry age could be partly explained by the “healthy worker effect” that, on the other hand, was not so clear in the US occupational Railroad cohort and absolutely absent in the Zrenjanin occupational cohort. More generally, the role of age at entry cannot be interpreted like other determinants since in multivariate models, it mainly represents an adjustment factor for the spread on age at entry examination (40 to 59 years).

ENE intake had an important predictive role. Sometimes it is interpreted as energy expenditure starting from the assumption of a metabolic balance, but in our case, its measurement was taken only based on food groups and nutrients. LIF, OLI, VEG and FRU intake plus the MP/ST ratio and the M/S ratio are among the food groups and ratios with the highest positive R values, which contrasted with the negative R values of ENE, HAF, THI, and INF (all inversely related to AD). The first group is also strongly related with the Mediterranean Diet, whose characteristics can also be seen in the positive role of the M/S and MP/ST ratios and the inverse association with THI and INF. In fact, INF was constructed on the basis of its positive associations with energy, fat, and saturated fat and its negative association with vegetables, fruit, olive oil, liquid fat, the sum of vegetable foods, and the ratio of vegetal/animal food [10]. By itself, a high inflammation index is a mediator between external causes and the initiation and development of many chronic diseases, and in this case, the food groups and nutrients directly or inversely associated with them may represent some of the external causes in different ways.

In general, the correlates of variables selected as significant in the univariate analysis should also be considered for interpretation. The most typical example is provided by the strong and significant role of LIF, which is an indicator of the Mediterranean diet but also a variable closely related with others typical for the Mediterranean diet, as seen from our correlation matrix. Incidentally, LIF has an R with AD of 0.70, and its components, i.e., olive oil and all seed oils, have R values of 0.63 and 0.17, respectively, suggesting that there is an advantage of combining them, although olive oil represents 84% and seed oils represent 16% of total liquid fat. A plausible takeaway message from these analyses is that several features of the Mediterranean diet are positively associated with longevity.

A few classic CVD risk factors never entered the list with significantly high Rs, not even blood pressure and serum cholesterol or smoking. One of the possible reasons might be the limited variance of mean blood pressure across the 16 cohorts (standard deviation equal to 4% of mean), while a larger but still small variance was found for serum cholesterol (standard deviation equal to 16% of mean). However, we know that serum cholesterol is predictive only of coronary heart disease (CHD), and this condition represents a substantial but not exceedingly high contribution to mortality, except in Finland. On the other hand, the association of serum cholesterol with 50-year CHD mortality was very large across the 16 cohorts; CHD mortality was higher than 10 per 1000 person/years in 5 of 16 cohorts, while it was definitely lower in the other 11 cohorts, and the proportion of CHD over all-cause mortality was greater than 25% only in the same 5 cohorts [18].

The coefficient of variation for smoking prevalence was also relatively low (14%), describing a limited variance at the ecologic level of this lifestyle habit across cohorts, probably obscuring its role in the association with AD. However, some findings about the role of traditional CVD risk factors have already been published from the Italian rural areas of the same study [19] and partly from the group of the 10 SCS cohorts that have reached death [20]. An inverse (although not significant) role versus AD of smoking habits and of systolic blood pressure was found at the ecological level, while the analysis on individuals indicated a direct association of vigorous physical activity and an adverse association of smoking habits, serum cholesterol, systolic blood pressure and heart rate. It is therefore clear that the same risk factors remain predictive of CVD and other conditions at the individual level and that the conclusion of this analysis cannot be directly applied to individuals.

A major problem of this analysis consisted of the difficult choice and handling of multivariate models and the need to exclude the linear multiple regression due to the small number of statistical units and due to the multicollinearity observed in the linear models. Beyond the technical limitation to comply with the small number of statistical units, each variable tends to be largely associated with the other variables either in a direct or inverse way, as clearly shown in the correlation matrix of Table 4. This means that each variable carries, indirectly, some information from the others.

The first problem was obviously impossible to solve, while for the second one, a partial solution was derived from the use, as a single covariate, of the Dietary Score derived from a PCA involving the 11 variables significant in the univariate analysis, producing a satisfactory outcome. It is of interest that the algebraic signs of factor scores of the PCA computed with the 11 major predictive variables were coherent with those found in the univariate analysis dealing with R and including the same variables. In fact, PCA is an *a posteriori* score procedure that does not include the end-point (that is AD) and is not influenced by the investigator opinions that often impinge upon selections if performed *a priori*. The relationship of Rs derived from univariate analysis and the factor scores of PCA showed their ability to segregate variables directly related to AD from those inversely so. Probably the most relevant finding of this analysis stays in the correlation between the Dietary Score (as a single determinant) and AD. Moreover, we used the Principal Component regression and the Ridge regression due to their specific characteristics and made the decision to select three groups of variables on the basis of an arbitrary *a priori* approach being, from this point of view, rather parsimonious. At the end, the findings were satisfactory and practically superposable with other results. Attempts to produce valuable models with six variables definitely failed.

All of these findings provide a clear lesson from ecological analyses comparing different cohorts. Possible determinants are more likely to be real predictive variables if their coefficient of variation is very high, say, more than 100%, as partly found in this analysis. Significant differences across the statistical units may not represent possible causes but only preliminary interpretations for differences. One should not infer that the same association could be found at the individual level but, if so, the fact represents a suggestion of a possible common causal role.

An ecological paradox finding was published in 1997 using the 25-year follow-up data of the SCS where the association of blood pressure with stroke mortality rates was inverse, while it was direct in analyses on individuals [21]. A possible explanation was identified in the fact that cohorts with high cholesterol and high blood pressure produced an excess of CHD deaths, while cohorts with low cholesterol and lower blood pressure produced an excess of stroke deaths, apparently due to the stronger role of serum cholesterol compared to that of high blood pressure for CHD mortality among cohorts.

An ecological analysis, partly based on the same dietary data and the same follow-up period of 50 years, was published in 2017 [17]: it showed that mortality from all causes was directly associated with energy and hard fats and inversely associated with vegetables, liquid fats, the MS ratio, and other complex food groups and ratios, partly in line with the present findings although using a different end-point (50-year all-cause death rate instead of 50-year AD). Incidentally, like in this paper, a strong component was energy intake, while the strongest food group was oils (liquid fats), with their Rs carrying opposite algebraic signs due to the different end-points.

It is clear that all of these observational findings do not solve the problem of the possible causality of eating habits that may need proper preventive trials. This accomplishment was recently reached by two Spanish trials where successes in primary and secondary prevention of major cardiovascular disease were obtained using some food components of the Mediterranean diet [22,23]. Despite this, it might be uncertain whether the same conclusions can be extended to longevity and AD, also in relation to technical issues dealing with extremely long trials.

The dispute on the role of saturated fat versus liquid fat seems never ending. Our findings, identifying LIF as the primary positive correlate of AD, align with the 2025 DGAC Scientific Report and the AHA news release [24,25]. However, they diverge from the final 2025–2030 DGA policy [26], which de-emphasizes the role of saturated fat, highlighting a critical contemporary conflict between long-term epidemiological data and current USA nutrition policy. However, a recent viewpoint of the problem is totally in line with the message of this analysis [27], and much debate is still ongoing [28]. With the limitation of having been conducted essentially within health profession settings, the controversy has recently been enriched by an additional element. The study just published online in the *Journal of the American College of Cardiology* [29] prospectively examined the association between diets characterized by different quantities and qualities of macronutrients, their metabolomic effects, and the risk of ischemic heart disease in 42,720 men in the Health Professionals Follow-Up Study (HPFS) (1986–2016), 64,164 women in the Nurses’ Health Study (NHS) (1986–2018), and 91,589 women in the NHSII (1991–2019), using dietary questionnaires aimed at defining the different origins and quality of foods (animal versus plant foods, whole grains versus refined carbohydrates, and other comparisons) rather than simply classifying diets as being low in saturated fat or carbohydrates. During 5,248,916 person-years, 20,033 cases of ischemic heart disease occurred, and the critical role of diet quality was highlighted, particularly when associated with fruits and vegetables rather than animal-derived foods, in reducing risk, since the “healthy” foods characteristic of such diets, much more than individual nutrients and their ratios, may exert beneficial health effects and significantly reduce coronary risk. These important individually obtained results align with the ecological ones presented in Table 6 of this study, obtained collectively using Principal Component and Ridge models in relation to LIF, FRU and VEG and determined as positively related to AD: this represents a clear allusion to the quality of a Mediterranean diet largely containing olive oil [10,17,22,23,25].

A frequently mentioned problem is bound to the possible changes in eating habits and nutrient contents of food during long follow-up periods. Re-analyses of the available food groups of each cohort after about 30 years of follow-up showed only slight changes that did not affect the ranks of the various nutrients across the cohorts [12,14,15]. A new dietary survey conducted after 25–30 years would cover no more than 30% of survivors aged 70 to 89 years, whose contribution to the association with AD would be rather limited.

We are unaware of other population studies that provided evidence on the relationship of risk factors and personal characteristics with AD in a long follow-up observation, and therefore, we are unable to quote any current references derived from previous investigations.

## 5. Conclusions

A group of 10 dietary nutrition variables were significantly ecologically associated with AD, and 6 of them could be tested in dedicated multivariate analyses. From the combined review of univariate and multivariate analyses, it appears that LIF (olive oils accounting in the largest proportion), FRU and VEG favour longevity, while hard fats and indicators of high intake of saturated and trans fats may play an adverse role. We can claim that around 50% of variance in cohort AD is explained by dietary habits. Similar findings were observed in an earlier publication using all-cause mortality as the end-point for the same follow-up duration [17]. On the other hand, traditional risk factors and personal characteristics did not contribute to explain the AD differences across the 16 study cohorts, likely due to their small coefficients of variation. We do not claim that traditional CVD risk factors are not associated with all-cause mortality or AD but simply that in the ecological approach, the priority in that association was found for the role of dietary nutrition measurements.

These findings underline the large contribution of eating habits in relation to longevity expressed by AD, but it should be recalled that the enrollment of the study cohorts was partly based on the hypothesized differences in habitual diets in relation to CHD occurrence [11] as a valuable basis for showing differences in outcome.

## Figures and Tables

**Table 1:** List of abbreviations and units of measurement.

	Unit of Measurement	Abbreviation
US Railroad cohort	----	US
East Finland cohort	----	EF
West Finland cohort	----	WF
Zutphen, the Netherlands	----	ZU
Crevalcore, Italy	----	CR
Montegiorgio, Italy	----	MO
Rome Railroad, Italy	----	RR
Dalmatia, Croatia-former Yugoslavia	----	DA
Slavonia, Croatia-former Yugoslavia	----	SL
Velika Krsna, Serbia-former Yugoslavia	----	VK
Zrenjanin, Serbia-former Yugoslavia	----	ZR
Belgrade, Serbia-former Yogoslavia	----	BE
Crete, Greece	----	KT
Corfu, Greece	----	CO
Tanushimaru, Japan	----	TA
Ushibuka, Japan	----	UB
Energy intake	calories per day	ENE
Liquid fat intake	g per day per 1000 calories	LIF
Hard fat intake	g per day per 1000 calories	HAF
Fruit intake	g per day per 1000 calories	FRU
Olive oil intake	g per day per 1000 calories	OLI
All vegetable food intake	g per day per 1000 calories	VEG
Monounsaturated fat/saturated fat intake	ratio	M/S
Mono plus polyunsturated fat/saturated plus trans-fat intake	ratio	MP/ST
DietaryThrombogenicity index	arbitrary units	THI
Dietary inflammation Index	arbitrary units	INF
Age	years	AGE

**Table 2:** Descriptive data of 50-year death rates and mean age at death in the 16 cohorts.

Cohort	Death Rates per 1000 Person/Year	Rank of Death Rate (*)	Age at Death Years	Rank of Age at Death (**)
US Railroad	37.3	8	75.1	8
East Finland	44.0	15	72.1	15
West Finland	40.4	13	73.1	13
Zutphen, the Netherlands	38.8	10	74.5	9
Crevalcore, Italy	39.1	11	74.5	10
Montegiorgio, Italy	36.2	6	76.3	4
Rome Railroad, Italy	34.4	3	77.9	3
Dalmatia, Croatia, former Yugoslavia	36.3	7	75.4	7
Slavonia, Croatia former Yugoslavia	45.3	16	69.7	16
Velika Krsna, Serbia, former Yugoslavia	39.9	12	73.8	11
Zrenjanin, Serbia, former Yugoslavia	43.1	14	72.9	14
Belgrade, Serbia, former Yugoslavia	31.1	2	77.9	2
Crete, Greece	31.1	1	80.4	1
Corfu, Greece	35.9	4	76.1	5
Tanushimaru, Japan	35.9	5	75.4	6
Ushibuka, Japan	38.6	9	73.6	12
Coefficient of variation (***)	4.1	-----	3.3	-----

(*) From the lowest to the highest. (**) From the highest to the lowest. (***) Standard deviation as % of mean.

**Table 3:** Simple linear correlation coefficients between significant variables and mean age at death plus PCA score coefficients for the same variables derived from PCA procedure. For meaning of abbreviations of variables, see Table 1.

Rank	Variable	R	R^2^	*p* Value	Partial Correlation	PCA Factor Scores
1	LIF	0.70	0.49	0.0024	0.80 (*)	−0.1255
2	OIL	0.63	0.40	0.0088	0.27	−0.1229
3	M/S	0.62	0.38	0.0104	0.31	−0.1320
4	INF	−0.62	0.38	0.0104	−0.43	0.1178
5	HAF	−0.61	0.37	0.0120	0.40	0.1138
6	ENE	−0.61	0.37	0.0120		0.0852
7	FRU	0.60	0.36	0.0140		−0.1084
8	MP/ST	0.59	0.35	0.0162		−0.1377
9	THI	−0.57	0.32	0.0212		0.1128
10	VEG	0.56	0.31	0.0240		−0.1323
11	AGE	−0.50	0.25	0.0486		0.0044

Partial correlation was computed only for each of the first 5 significant determinants versus AD, while the 4 other significant determinants play the role of confounding variables. (*) *p* value of partial correlation ≤ 0.05.

**Table 4:** Correlation matrix (for R) of 11 entry variables significantly related with mean age at death. For meaning of abbreviations of variables, see Table 1.

	AGE	ENE	FRU	OLI	LIF	HAF	VEG	INF	M/S	THI	MP/ST
**AGE**	1.00	0.21	−0.22	0.04	−0.12	−0.02	0.09	0.11	0.02	−0.08	0.08
**ENE**		1.00	−0.40	−0.19	−0.23	**0.55**	**−0.55**	**0.74**	−0.30	**0.80**	−0.46
**FRU**			1.00	**0.69**	**0.72**	−0.42	**0.75**	**−0.60**	**0.73**	−0.33	**0.66**
**OLI**				1.00	**0.98**	**−0.54**	**0.72**	**−0.61**	**0.96**	−0.46	**0.86**
**LIF**					1.00	**−0.60**	**0.72**	**−0.61**	**0.96**	−0.47	**0.87**
**HAF**						1.00	**−0.68**	**0.62**	**−0.63**	**0.83**	**−0.79**
**VRG**							1.00	**−0.70**	**0.82**	**−0.75**	**0.89**
**INF**								1.00	**−0.65**	**0.70**	**−0.75**
**M/S**									1.00	**−0.58**	**0.94**
**THI**										1.00	**−0.77**
**MP/ST**											1.00

Values of R ≥ 0.50 correspond to *p* values < 0.05 (in bold).

**Table 5:** Mean levels in 16 cohorts of 11 variables significantly associated with mean age at death (through R). All items are given as grams per day, except ENE (energy), which is given as calories per day. For meaning of abbreviations of variables, see Table 1.

**Cohort**	**LIF**	**OLI**	**M/S**	**INF**	**HAF**	**ENE**	**FRU**	**M/PST**	**THI**	**VEG**	**AGE**
**US**	1.3	0.0	0.88	−0.23	12.9	2326	100.2	1.12	49.8	281.6	49.4
**EF**	0	0.0	0.61	1.46	27.1	3577	11.2	0.69	76.6	244.1	48.8
**WF**	0	0.0	0.68	2.25	21.2	3440	9.9	0.68	64.7	261.3	49.9
**ZU**	0	0.0	9.30.81	2.70	27.4	2922	28.1	0.81	53.4	284.7	49.5
**CR**	11.4	9.3	1.32	2.24	15.8	3432	55.7	1.32	49.7	263.1	49.2
**MO**	8.6	8.6	1.60	0.61	14.3	2791	10.0	1.60	29.7	305.3	49.0
**RR**	17.5	17.5	1.93	−0.23	2.4	2455	61.1	1.93	26.1	246.2	48.3
**DA**	22.5	22.5	1.97	0.30	5.3	3201	1.9	1.97	36.9	311.8	50.4
**SL**	2.1	2.1	1.18	2.98	16.3	3816	0.3	1.18	66.3	245.3	50.4
**VK**	1.5	0.0	0.81	2.17	7.1	3388	0.3	0.81	46.6	293.4	49.7
**ZR**	3.7	0.0	1.19	1.73	13.5	3256	56.8	1.19	52.8	338.1	49.0
**BE**	10.1	0.0	0.95	1.39	9.7	2870	52.2	0.95	51.8	267.6	47.0
**KT**	35.0	35.0	2.96	0.11	0.0	2712	171.1	2.96	25.0	508.9	49.0
**CO**	28.9	28.9	2.85	−1.10	0.0	2540	178.1	2.85	19.8	540.9	49.7
**TA**	1.3	0.0	0.99	0.46	0.0	2243	11.6	0.99	9.3	402.6	50.1
**UB**	3.1	0.0	1.23	0.60	0.0	2267	18.5	1.23	13.2	371.9	49.5
Coef Var (*)	113.8	147.2	50.8	102.8	88.4	28.2	111.6	46.9	45.7	26.1	1.60

(*) Coefficient of variation = standard deviation as % of average.

**Table 6:** Principal Component regression and Ridge regression models predicting age at death with 3 or 2 different arbitrarily selected variables. For meaning of abbreviations of variables, see Table 1.

**Principal Component Model with 3 Variables Directly Related to AD in Univariate Analysis**				
Variables	Coefficient	Standard error	Standardized coefficient	Variance Inflation Factor
Intercept	72.5990			
LIF	0.1319	0.0722	0.5766	2.4761
FRU	0.0039	0.0723	0.0863	0.7449
VEG	0.0028	0.0047	0.0971	0.6701
**Analysis of variance with 1 component omitted, *p* = 0.0293**			**R between observed and estimated AD = 0.72**	
**Principal Component model with 3 variables inversely related to AD in univariate analysis**				
Variables	Coefficient	Standard error	Standardized coefficient	Variance Inflation Factor
Intercept	77.1954			
HAF	−0.0303	0.0529	−0.1104	0.7834
THI	−0.235	0.0154	−0.4283	1.8032
INF	−0.9141	0.6243	−0.1827	0.3037
**Analysis of variance with 1 component omitted, *p* = 0.0.713**			**R between observed and estimated AD = 0.66**	
**Principal Component model with 2 variables related to AD in opposite way**				
Variables	Coefficient	Standard error	Standardized coefficient	Variance Inflation Factor
Intercept	75.1738			
LIF	0.0885	0.0255	0.3870	0.3121
HAF	−0.1062	0.0307	−0.3870	0.3121
**Analysis of variance with 1 component omitted, *p* = 0.0143**			**R between observed and estimated AD = 0.69**	
**Ridge model with 3 variables directly related to AD in univariate analysis**				
Variables	Coefficient	Standard error	Standardized coefficient	Variance Inflation Factor
Intercept	73.1870			
LIF	0.1322	0.0721	0.5783	2.4739
FRU	0.0074	0.0147	0.1654	2.6981
VEG	0.0005	0.0095	0.0163	2.7078
**Analyis of variance: *p* = 0.0282**			**R between observed and estimated AD = 0.72**	
**Ridge model with 3 variables inversely related to AD in univariate analysis**				
Variables	Coefficient	Standard error	Standardized coefficient	Variance Inflation Factor
Intercept	77.1722			
HAF	−0.0324	0.1090	−0.1179	3.3238
THI	−0.0223	0.0557	−0.1737	3.9620
INF	−0.9182	0.6520	−0.4302	1.9672
**Analysis of variance = 0.0713**			**R between observed and estimates AD = 0.66**	
**Ridge model with 2 variables related to AD in opposite way**				
Variables	Coefficient	Standard error	Standardized coefficient	Variance Inflation Factor
Intercept	74.0749			
LIF	0.1398	0.0550	0.6117	1.5684
HAF	−0.0446	0.0660	0.6123	1.5684
**Analysis of variance with *p* = 0.0085**			**R between observed and estimated AD = 0.72**	

## Data Availability

The data used in this analysis are derived from the Seven Countries Study. Due to internal rules, the data and computing codes are not publicly available, although specific requests for dedicated analyses may be evaluated by the internal Coordinating Committee.

## Appendix A

**Table A1:** List of 58 baseline variables tested for the association with age at death.

Type of Variable	Variables
13 traditional CVD risk factors	age, socio-economic status, body mass index, systolic blood pressure, diastolic blood pressure, serum cholesterol, subscapular skinfold, heart rate, cigarette per day, smoker prevalence, sedentary physical activity, moderate physical activity, vigorous physical activity
20 simple food groups	bread, cereals, potatoes, vegetables, legumes, fruit, meat, butter, milk, cheese, eggs, margarine, lard, fish, olive oil, n6-poly-oil, sugar, pastries, alcohol, wine
4 combined food groups	hard fat, liquid fat, sum of vegetable food, sum of animal food,
13 nutrients	energy, protein, sucrose, natural sugar, added sugar, mono-di-saccharides, starch, all carbohydrates, fat, saturated fat, monounsaturated fat, polyunsaturated fat, trans-fat
8 ratios of foods or nutrients, and Dietary Scores	ratio animal food/vegetable food, monounsaturated fat/saturated fat, monounsaturated + polyunsaturated fat/saturated + trans fat, polyunsaturated fat/saturated fat, Mediterranean Adequacy Index, dietary inflammation score, dietary atherogenicity index, dietary thrombogenicity index
